# Abatacept decreases disease activity in a absence of CD4^+^ T cells in a collagen-induced arthritis model

**DOI:** 10.1186/s13075-015-0731-1

**Published:** 2015-08-20

**Authors:** Diahann TSL Jansen, Hanane el Bannoudi, Ramon Arens, Kim LL Habets, Marjolijn Hameetman, Tom WJ Huizinga, Jeroen N. Stoop, René EM Toes

**Affiliations:** Department of Rheumatology, Leiden University Medical Centre, PO Box 9600, 2300 RC Leiden, the Netherlands; Department of Immunohematology and Blood Transfusion, Leiden University Medical Centre, PO Box 9600, 2300 RC Leiden, the Netherlands

## Abstract

**Introduction:**

Abatacept is a fusion protein of human cytotoxic T-lymphocyte–associated protein (CTLA)-4 and the Fc portion of human immunoglobulin G1 (IgG1). It is believed to be effective in the treatment of rheumatoid arthritis by inhibiting costimulation of T cells via blocking CD28–B7 interactions as CTLA-4 binds to both B7.1 (CD80) and B7.2 (CD86). However, the interaction of CD28 with B7 molecules is crucial for activation of naive cells, whereas it is unclear whether the action of already activated CD4^+^ T cells, which are readily present in established disease, also depends on this interaction. The aim of this study was to determine whether the mode of action of abatacept depends solely on its ability to halt T cell activation in established disease.

**Methods:**

Arthritis was induced in thymectomized male DBA/1 mice by immunisation with bovine collagen type II. The mice were subsequently depleted for CD4^+^ T cells. Abatacept or control treatment was started when 80 % of the mice showed signs of arthritis. Arthritis severity was monitored by clinical scoring of the paws, and anti-collagen antibody levels over time were determined by enzyme-linked immunosorbent assay.

**Results:**

Treatment with abatacept in the absence of CD4^+^ T cells resulted in lower disease activity. This was associated with decreasing levels of collagen-specific IgG1 and IgG2a antibodies, whereas the antibody levels in control or CD4^+^ T cell–depleted mice increased over time.

**Conclusions:**

These results show that abatacept decreased disease activity in the absence of CD4^+^ T cells, indicating that the mode of action of abatacept in established arthritis does not depend entirely on its effects on CD4^+^ T cell activation.

## Introduction

Rheumatoid arthritis (RA) is a chronic inflammatory autoimmune disease affecting the joints in approximately 1 % of the world’s population [1, 2]. Patients with RA can be treated with non-steroidal anti-inflammatory drugs (NSAIDs) or with disease-modifying anti-rheumatic drugs (DMARDs). NSAIDs can alleviate disease symptoms, but they do not impede the underlying inflammatory events or inhibit joint destruction; however, DMARDs do affect the disease process in all these respects [3]. Abatacept, a fusion protein of human cytotoxic T-lymphocyte–associated protein (CTLA)-4 and the Fc portion of human immunoglobulin G1 (IgG1), is a (biologic) DMARD and is an effective therapy for established RA [4, 5]. It is believed to be effective by blocking the costimulation of T cells through disruption of CD28–B7 interactions as CTLA-4 binds to B7.1 (CD80) and B7.2 (CD86) on antigen-presenting cells (APCs) [6].

CTLA-4-Ig has been tested in the collagen-induced arthritis (CIA) model in mice and rats as a preventative treatment and on the first day of clinical onset, resulting in lower clinical scores and reduced joint damage [7–9]. However, abatacept is used to treat RA patients in whom anti–tumour necrosis factor (anti-TNF) treatment has failed. It is likely that, in this phase of disease, the underlying autoimmune response is fully matured. Likewise, it is conceivable that the action of abatacept does not fully depend on its ability to inhibit T cell responses, as fully developed T cell responses are less dependent on CD28 costimulation. Indeed, CD28–B7 interactions are important for the activation of naive T cells, but this is less well established for the activation of memory CD4^+^ T cells [10]. CD28-B7 costimulation of memory CD4^+^ T cells has been described as disturbing interleukin (IL)-2 production and proliferation; however, production of other cytokines and expression of activation markers CD25 and CD69 are not affected, indicating an incomplete dependence on this pathway [11]. Therefore, it is of interest to study the effect of abatacept in the established phase of arthritis models, as it is more similar to the human situation with respect to the developmental phase of the underlying autoimmune response. In addition, much can be learned about the pathogenesis of human disease by understanding the mode of action of therapeutic interventions. The latter is exemplified through the use of anti-TNF or IL-6R blocking agents, for example, showing the pivotal role of these cytokines in inflammation. Nonetheless, the exact mode of action of several DMARDs used in RA treatment, such as methotrexate or sulfasalazine, is still largely unclear.

Recently, researchers compared anti-TNF treatment (adalimumab) with abatacept in a head-to-head study revealing similar efficacy in time based on clinical, functional and radiographic outcomes [12]. Intriguingly, anti-TNF therapy is thought to have a quick mode of action, as it directly inhibits inflammation by blocking TNF, whereas abatacept is thought to be effective after a longer time period, as the effect of costimulation blockade does not become apparent directly. Consequently, similar efficacy of adalimumab and abatacept indicates a different mode of action of abatacept in addition to costimulation blockade. Therefore, in the present study, we investigated whether the mode of action of abatacept depends solely on its ability to halt T cell activation. We report a decrease in disease progression and activity after abatacept treatment in the absence of CD4^+^ T cells, indicating that the mode of action of abatacept in established arthritis does not depend entirely on its effects on CD4^+^ T cell activation.

## Methods

### Mice

Male DBA/1 mice were obtained from our own breeding colony (originally obtained from Charles River Laboratories, Wilmington, Massachusetts, USA). Thymectomized DBA/1 mice were purchased from Harlan Laboratories (Boxmeer, the Netherlands). All mice were housed under specific pathogen-free conditions in individually ventilated cages at the animal facility of Leiden University Medical Centre, Leiden, the Netherlands. All experiments were performed in accordance with Dutch national legislation and approved by the ethics committee of the Animal Experiments Committee of Leiden University (approval numbers 11085 and 12217).

### Induction of CIA and evaluation of arthritis

CIA was induced in 8–10-week-old male DBA/1 mice as described elsewhere [13]. A clinical score was assigned based on a scoring protocol in which each swollen or red phalanx was given 0.5 point and 1 point per toe. A red or swollen knuckle was given 1 point, and a red or swollen footpad and a swollen ankle and/or wrist were given 5 points. The maximum score for each paw was 15 points, resulting in a maximum possible score of 60 points per mouse. Disease progression was monitored for a maximum of 90 days after induction of CIA. Change in clinical score was calculated by subtracting the clinical score at the start of treatment for every scoring time point after start of treatment until the end of follow-up to correct for the difference in clinical score at the start of treatment, as the mice did not develop arthritis at the same time.

### Treatment

Treatment was started when 80 % of the mice showed signs of arthritis. The mice were randomised to the different treatment groups according to their scores to ensure that the mean clinical score of all groups was comparable at the start of treatment. On day 0 of treatment, 100 μg of GK1.5 (rat anti-mouse CD4 monoclonal antibody [mAb]) was administered intraperitoneally to the mice that underwent CD4^+^ T cell depletion to acquire CD4 depletion at the start of treatment. This was continued weekly until the end of the experiment. For the different treatment regimens, mice received intraperitoneal injections of 500 μl of phosphate-buffered saline (PBS), 100 μg of GK1.5, 1 mg of abatacept (Bristol-Myers Squibb, Princeton, NJ, USA), 100 μg of GK1.5 in combination with 1 mg of abatacept, 1 mg of isotype for abatacept (Roche, Basel, Switzerland) or 1 mg of isotype in combination with 100 μg of GK1.5 on days 1, 3, 5, 8, 12 and 19.

### Evaluation of CD4^+^ T cell counts

To confirm that CD4^+^ T cell depletion after GK1.5 treatment was complete, blood was collected on days 0, 12, 22 and 35 and at the end of the experiment. The blood was lysed and subsequently stained with CD3 PerCP-Cy5.5 (clone 145-2C11), CD4 fluorescein isothiocyanate (clone RM4-4), CD8 allophycocyanin (Ly-2, clone 53–6.7), all from BD Pharmingen (San Diego, CA, USA); and anti-mouse CD45 eFluor 450 (clone 30-F11; eBioscience, San Diego, CA, USA). All samples were evaluated by using a BD LSRFortessa cell analyser (BD Biosciences, San Jose, CA, USA) and analysed using BD FACSDiva software (BD Biosciences) and FlowJo version 7.6.5 software (Tree Star, Ashland, OR, USA).

### Measurement of serum antibodies by enzyme-linked immunosorbent assay

Anti–collagen type II and total IgG antibody levels were determined as described elsewhere [14]. In short, Nunc MaxiSorp plates (Thermo Scientific, Waltham, MA, USA) were coated with 2 μg/ml bovine collagen type II (Chondrex, Redmond, WA, USA) or 3 μg/ml murine collagen type II (Chondrex) for antigen-specific antibodies or with 0.5 μg/ml goat anti-mouse IgG (SouthernBiotech, Birmingham, AL, USA) for total antibodies. IgG, IgG1 and IgG2a were detected using goat anti-mouse IgG–horseradish peroxidase (HRP), goat anti-mouse IgG1-HRP and goat anti-mouse IgG2a-HRP, respectively (all from SouthernBiotech). Enzyme activity was visualised using 2,2′-azino-bis(3-ethylbenzothiazoline-6-sulphonic acid. Serial dilutions from pooled sera of arthritic mice were used as a standard to calculate arbitrary units.

### Measurement of supernatant antibody titres

After sacrifice bone marrow and spleen cells were isolated and then 200,000 cells per well were cultured in Iscove’s modified Dulbecco’s medium (Lonza BioResearch, Basel, Switserland) containing 10 % foetal calf serum (Gibco; Life Technologies, Carlsbad, CA, USA), GlutaMAX, penicillin, streptomycin (Invitrogen, Carlsbad, CA, USA) and 2-mercaptoethanol. After 7 or 14 days of culture, the supernatant was harvested and total IgG levels were determined by enzyme-linked immunosorbent assay (ELISA).

### Statistical analysis

Statistical analysis was performed using GraphPad Prism version 5 (GraphPad Software, La Jolla, CA, USA). The abatacept and CD4 depletion combination-treated group and the control group were compared using Student’s *t* test or the Mann–Whitney *U* test as appropriate according to data distribution. *P* values <0.05 were considered to be significant.

## Results

### Abatacept decreased disease activity in mice depleted of CD4^+^ T cells by GK1.5

**Fig. 1:**
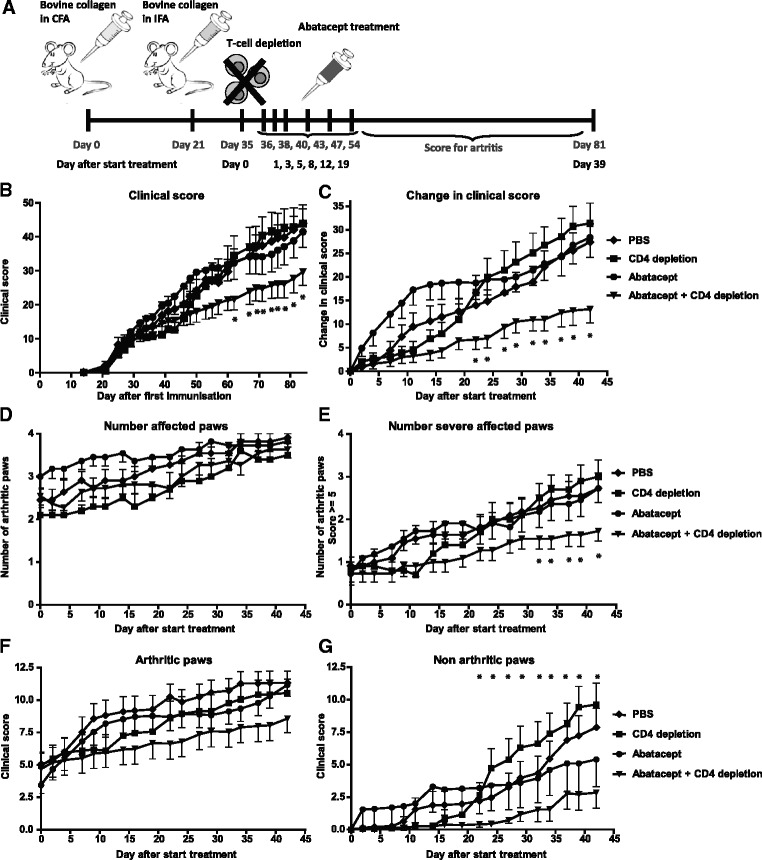
Abatacept decreased disease activity in mice depleted of CD4^+^ T cells by GK1.5. **a** Collagen-induced arthritis was induced in male DBA/1 mice. When 80 % of the mice showed signs of arthritis, treatment was started. One day before the start of treatment, CD4^+^ T cells were depleted by intraperitoneal administration of GK1.5, and the depletion was continued until the end of follow-up. Treatment was administered by intraperitoneal injection of either PBS (*diamonds*), GK1.5 (CD4 depletion) (*squares*), abatacept (*circles*) or a combination of GK1.5 and abatacept (*triangles*). The mice were scored three times per week for inflammation in the paws to monitor disease progression. **b** Clinical scores over time of the different treatment groups. **c** Changes in clinical scores starting from the day treatment was initiated in the different treatment groups. **d** Number of affected paws per treatment starting from the day treatment was initiated. **e** Number of affected paws with a clinical score of 5 or higher (considered to be severely affected paws) per treatment starting from the day treatment was initiated. **f** Clinical scores of paws that showed signs of arthritis at the start of treatment and extending over time, per treatment. **g** Clinical scores of paws that did not show signs of arthritis at the start of treatment, per treatment, over time. Values are mean ± SEM (n=11 per treatment group). Statistical analysis was performed using Student’s *t* test. *P < 0.05 abatacept + CD4 depletion vs CD4 depletion. *CFA* complete Freund’s adjuvant, *IFA* incomplete Freund’s adjuvant, *PBS* phosphate-buffered saline

CTLA-4-Ig treatment has been used in the CIA model as a preventative intervention [7–9]. However, CTLA-4-Ig has not been tested in established disease, where most of the disease-associated T cells are thought to be already activated or to have differentiated into memory T cells. Hence, it is not known whether its mode of action is also mediated through T cell inhibition in this disease phase. To investigate this mode of action, CIA was induced and treatment was started when 80 % of the mice showed signs of arthritis. One day before the start of treatment, CD4^+^ T cells were depleted by intraperitoneal administration of the CD4^+^ T cell–depleting mAb GK1.5, and GK1.5 treatment was continued weekly until the end of follow-up. Intriguingly, mice treated with the combination of CD4 depletion and abatacept showed a significant decrease in disease activity compared with the mice treated with GK1.5 only or with PBS control (Fig. 1b,c). In contrast, CD4 depletion only did not significantly alter arthritis development compared with the control group (Fig. 1b,c). Abatacept treatment in combination with CD4 depletion did not result in a lower number of affected paws; however, combination treatment did reduce the number of severely affected paws (clinical score ≥5) (Fig. 1d,e). In addition, novel paws that developed inflammation after the start of therapy displayed a lower disease score. Thus, abatacept treatment did not prevent arthritis development in unaffected joints, but it did reduce clinical scores of affected joints (Fig. 1f,g). Similar results were also obtained in an independent replication experiment (data not shown).

**Fig. 2:**
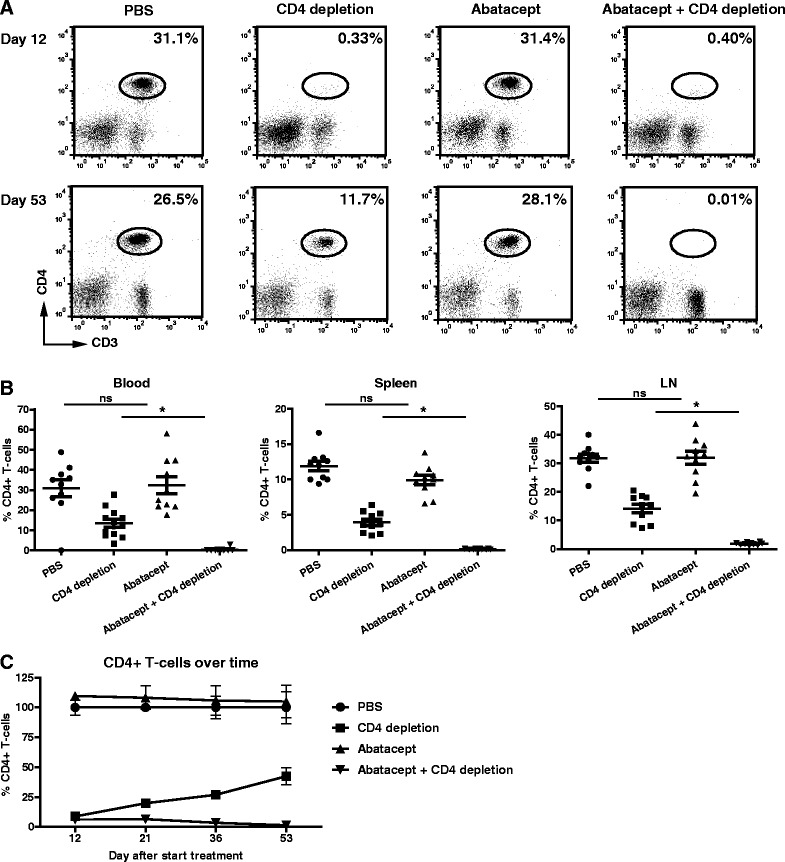
Incomplete CD4^+^ T cell depletion by GK1.5 over time. The presence of CD4^+^ T cells in the blood was monitored over time using flow cytometry. Blood was collected by making tail incisions during the experiment or by cardiac puncture at the of sacrifice. After red blood cell lysis, blood mononuclear cells were cell-surface stained for CD45, CD3, CD4 and CD8. Cells were gated on CD45 and subsequently on CD3 and CD4. **a** Dot plots of representative mice of each treatment group on day 12 and day 53 (end of experiment) after the start of treatment. **b** Summary of the percentages of CD4^+^ T cells per treatment at the end of follow-up is depicted for the blood, spleen and inguinal lymph node (LN). Each symbol represents 1 mouse. **c** Summary of CD4^+^ T cells in the blood over time as a percentage of the phosphate-buffered saline (PBS)-treated group. Values are mean ± SEM (n=11 per treatment group). Statistical analysis was performed using Student’s *t* test. *P < 0.05 abatacept + CD4 depletion vs CD4 depletion. *ns* not significant

To confirm complete CD4^+^ T cell depletion, CD4^+^ T cell frequencies were evaluated in the blood of GK1.5-treated mice. On day 12 after the start of treatment, CD4^+^ T cells were correctly depleted as expected. However, on day 53, CD4^+^ T cell depletion was not complete anymore. In contrast, mice receiving the combination of abatacept and CD4 depletion were still depleted of CD4^+^ T cells (Fig. 2a). This was also the case in the spleen and inguinal lymph node (Fig. 2b). Examining the frequency of CD4^+^ T cells over time indicated that, from day 12 onwards, the CD4^+^ T cell frequencies gradually increased until the end of the experiment. Nonetheless, over time, CD4^+^ T cell frequencies were still significantly lower than in the groups that did not receive GK1.5 (Fig. 2c). In contrast, CD4^+^ T cells in the mice receiving the combination of GK1.5 and abatacept treatment remained depleted (Fig. 2c), conceivably as a result of the prevention of the development of an anti-rat antibody response against GK1.5 by abatacept. Together, these results show that abatacept is also effective in situations where CD4^+^ T cell numbers are greatly reduced, suggesting a T cell–independent effect of abatacept that inhibits the progression of arthritis.

### Abatacept decreased disease activity in thymectomized mice depleted of CD4^+^ T cells

**Fig. 3:**
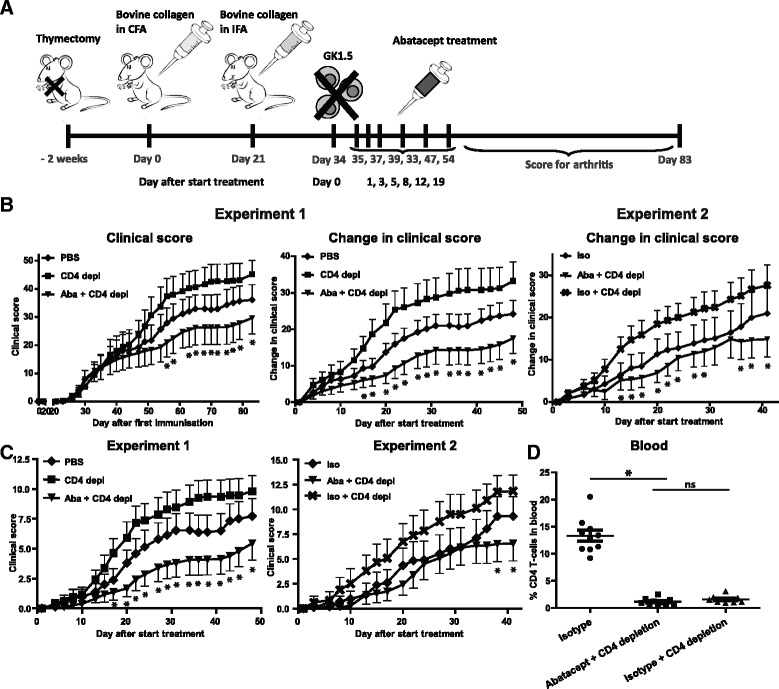
Abatacept decreased disease activity in thymectomized mice depleted of CD4^+^ T cells. **a** Collagen-induced arthritis was induced in male DBA/1 mice 2 weeks after they were thymectomized. When 80 % of the mice showed signs of arthritis, treatment was started. One day before the start of treatment, CD4^+^ T cells were depleted by intraperitoneal administration of GK1.5 and then the depletion was continued until the end of follow-up. Treatment was administered by intraperitoneal injection of PBS (*diamonds*), GK1.5 (*squares*; CD4 depl) or the combination of GK1.5 and abatacept (*triangles*; Aba + CD4 depl). The mice were scored three times per week for inflammation in the paws to monitor disease progression. **b** Clinical scores and changes in clinical scores starting from the day treatment was initiated in the different treatment groups (experiment 1; n=10 per treatment group). The same experiment was independently repeated in another 10 mice per treatment group, and an isotype for abatacept was used as control treatment (*diamonds*; iso) and in combination with CD4 depletion (*cross symbols*; iso + CD4 depl) (experiment 2). Changes in clinical scores starting from the day treatment was initiated are depicted. **c** The clinical scores of the paws that did not show signs of arthritis at the start of treatment are depicted for experiments 1 and 2. **d** The frequency of CD4^+^ T cells in blood at the end of follow-up was determined by flow cytometry. Abatacept-only treatment is not depicted to improve the readability of the graphs. Values are mean ± SEM. Statistical analysis was performed using Student’s *t* test. *P < 0.05 abatacept + CD4 depletion vs control group. *CFA* complete Freund’s adjuvant, *IFA* incomplete Freund’s adjuvant, *ns* not significant, *PBS* phosphate-buffered saline

Although the data presented above point to a CD4^+^ T cell–independent effect of abatacept in the treatment of arthritis, they do not show such effects in a conclusive manner for CD4^+^ T cells returned after initial depletion. To ascertain that the CD4^+^ T cells were completely depleted during the treatment, a more stringent method of CD4^+^ T cell depletion was implemented. CIA was induced in mice that were thymectomized at 6 weeks after birth. After immunisation with collagen type II and development of arthritis, CD4^+^ T cell depletion was performed using GK1.5. Because the mice were thymectomized, no new T cells could reappear in CD4^+^ T cell–depleted mice. Again, we observed that treatment with abatacept resulted in reduced disease activity in CD4^+^ T cell–depleted mice (Fig. 3b). Likewise, a reduced clinical score for paws that were not arthritic at the start of treatment was observed (Fig. 3c). We also noted a reduced number of severely affected paws, but abatacept treatment did not prevent arthritis development in joints not affected at the start of therapy (data not shown). Abatacept-only treatment did not modulate the clinical score compared with PBS treatment (data not shown). To confirm that CD4^+^ T cells were completely depleted, CD4^+^ T cell frequencies were monitored over time by flow cytometry. Contrary to treatment with GK1.5 only (Fig. 2), thymectomy in combination with GK1.5 treatment resulted in complete depletion of CD4^+^ T cells in mice that received CD4 depletion treatment (alone or in combination with abatacept) (Fig. 3d). These results indicate that abatacept treatment results in decreased disease activity in the absence of CD4^+^ T cells.

### Reduced antibody levels after treatment with abatacept in the absence of CD4^+^ T cells

**Fig. 4:**
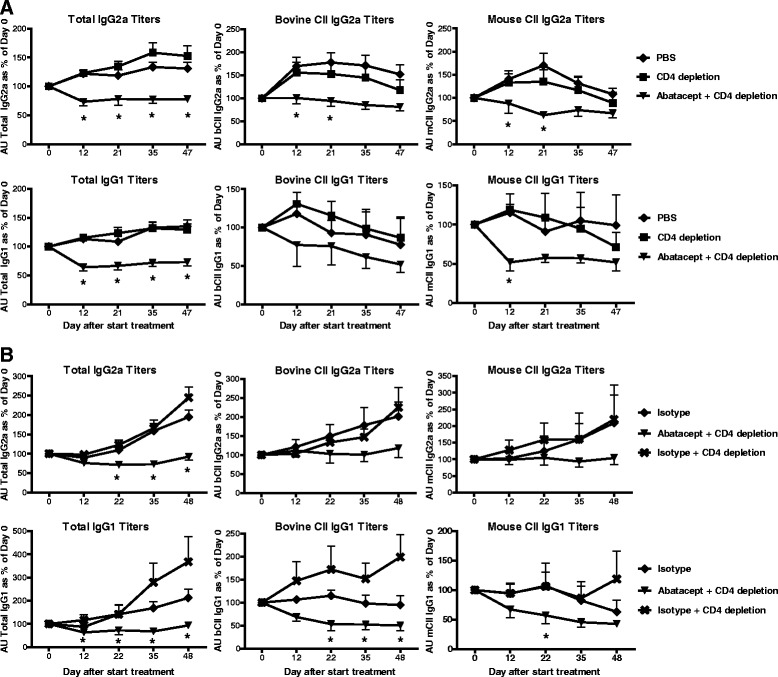
Reduced antibody levels after treatment with abatacept in the absence of CD4^+^ T cells. Antibody levels were determined by enzyme-linked immunosorbent assay over time. Serum samples were collected on days 0, 12, 21, 22, 35 and 47/48 after the start of treatment, and total IgG2a and IgG1 levels were determined in the thymectomized mice. In addition, bovine and mouse collagen type II–specific IgG2a and IgG1 levels were determined. **a** Levels are depicted as percentages of day 0. **b** An independent experiment including the isotype for abatacept. Abatacept-only treatment is not depicted to improve the readability of the graphs, but it showed results comparable to those for abatacept + CD4 depletion. Levels are depicted as percentages of day 0. Values are mean ± SEM (n=10 per treatment group). Statistical analysis was performed using the Mann–Whitney *U* test. *P < 0.05 abatacept + CD4 depletion vs control group. *AU* arbitrary units, *bCII* bovine collagen type II, *CII* collagen type II, *IgG* immunoglobulin G, *mCII* mouse collagen type II, *PBS* phosphate-buffered saline

Collagen type II–specific antibodies play a crucial role in the development of CIA [15]. Therefore, collagen type II–specific and total IgG levels were determined by ELISA over time in sera of the thymectomized mice. Treatment with abatacept in the absence of CD4^+^ T cells resulted in decreased total IgG2a levels over time compared with control treatment (Fig. 4). More importantly, decreases in bovine collagen type II (immunisation antigen) and murine collagen type II (autoantigen) levels were also detected after treatment with abatacept and GK1.5 (Fig. 4). This result was not specific for the IgG2a isotype, as decreased IgG1 levels were observed as well (Fig. 4). Together, these results indicate that abatacept treatment leads to reductions in disease activity and collagen-specific antibody levels in the absence of CD4^+^ T cells.

**Fig. 5:**
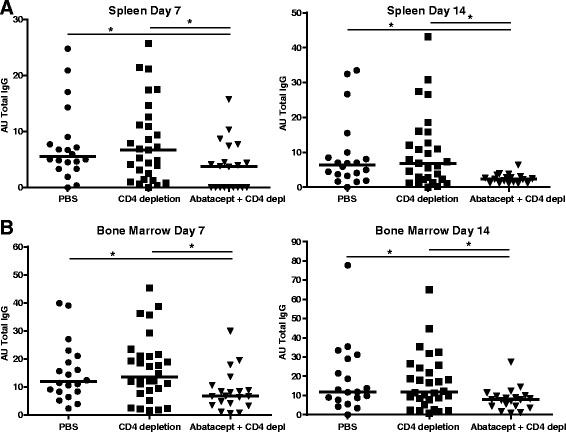
Abatacept decreased antibody levels detected in supernatants of ex vivo spleen and bone marrow cells in the absence of CD4^+^ T cells. After the animals were sacrificed, spleen and bone marrow cells were collected and cultured without stimulation for 7 or 14 days and then IgG production was measured in the supernatants by enzyme-linked immunosorbent assay. **a** Summary of the IgG levels detected in supernatants of spleen cells collected in experiments 1 and 2. The IgG levels of the phosphate-buffered saline (PBS)- and isotype-treated mice are combined and depicted as ‘PBS’, and the CD4 depletion and combination of isotype- and CD4 depletion–treated groups are combined and depicted as ‘CD4 depletion’. **b** Summary of the IgG levels detected in supernatants of bone marrow cells cultured for 7 or 14 days in experiments 1 and 2. Statistical analysis was performed using the Mann–Whitney *U* test. *P < 0.05 abatacept + CD4 depletion vs control group. *AU* arbitrary units, *IgG* immunoglobulin G

### Reduced antibody levels in supernatant of ex vivo cultured spleen and bone marrow cells after treatment with abatacept in the absence of CD4^+^ T cells

The data presented above suggest that abatacept treatment could directly affect the number and/or activity of antibody-secreting B cells. To study whether abatacept in addition to the inhibition of costimulation of T cells also affected the antibody-producing capacity of spleen- and bone marrow–derived B cells, we next isolated spleen and bone marrow cells from treated animals. Total IgG levels in the supernatants of spleen and bone marrow cells cultured ex vivo, but not stimulated, were subsequently analysed by ELISA. Spleen cells of mice treated with abatacept and CD4 depletion produced lower IgG levels after 7 and 14 days of culture than did spleen cells of mice that received only CD4 depletion (Fig 5a), although the percentages of B cells and plasma cells, as analysed by flow cytometry, were comparable between the different treatment groups (data not shown). This reduction in IgG production was also observed in the supernatants of cultured bone marrow cells (Fig 5b), indicating a loss of antibody-producing capacity after abatacept treatment in the absence of CD4^+^ T cells.

## Discussion

Abatacept is an effective treatment in RA and is thought to block costimulation of T cells by inhibiting CD28–B7 interactions as abatacept binds to both B7.1 and B7.2 [6]. The interaction of CD28 with both B7 molecules is crucially important for the activation of naive T cells, whereas its role in the activation of already activated and/or memory CD4^+^ T cells is less clear. As such CD4^+^ T cells are readily present in established disease, we investigated whether abatacept is still effective in the absence of CD4^+^ T cells in the established disease phase of the CIA model. Our study revealed that abatacept treatment is able to decrease disease activity in the absence of CD4^+^ T cells, indicating that the mode of action mediated by abatacept in CIA does not depend solely on its ability to block costimulation of T cells. In addition, abatacept treatment is capable of reducing collagen-specific and total antibody levels in a T cell–independent setting.

To evaluate the mode of action of abatacept in established disease, CD4^+^ T cells were depleted using the rat anti-mouse CD4 antibody GK1.5. Remarkably, after 12 days of depletion, the CD4^+^ T cells gradually reappeared in mice treated with GK1.5 only. Interestingly, the CD4^+^ T cells remained properly depleted in mice treated with a combination of abatacept and GK1.5. This observation is most likely explained by the notion that mice treated with GK1.5 develop an anti-rat antibody response that ultimately neutralises the CD4-depleting antibodies. This phenomenon illustrates that abatacept is also capable of blocking costimulation and thereby the activation of naive T cells, preventing the development of the anti-rat antibody response. Therefore, the depletion of CD4^+^ T cells by GK1.5 in mice treated with abatacept resulted in the complete and sustained depletion of CD4^+^ T cells until the end of follow-up. For this reason, we also depleted CD4^+^ T cells in thymectomized mice to prevent the reappearance of new T cells when the GK1.5 treatment became less effective, and we obtained similar results.

Our results indicate that CD4^+^ T cells are not required for disease progression once arthritis is established, because the mice treated with only GK1.5 showed comparable disease progression or a trend towards more severe disease progression compared with the control groups. These observations are in line with the observation by Morgan et al., who reported that lethal irradiation of mice with CIA, followed by syngeneic bone marrow transplantation, resulted in continuation of the disease even though the T cells were depleted [16]. Thus, CD4^+^ T cells are not required for arthritis progression once the disease clinically manifests in the CIA model. Our presented results were obtained in a mouse model; however, it is not known whether abatacept has a direct effect on other cell types in addition to T cells in humans. Nonetheless, in a recent study of RA in which researchers compared anti-TNF and abatacept treatment head to head, comparable efficacy was observed based on clinical, functional and radiographic outcomes [12]. Intriguingly, no difference in the rate of response was noted, as similar improvements were observed over time. As one could speculate that a T cell–targeting drug would require more time than a TNF inhibitor to manifest its beneficial effects, this observation could be compatible with the notion that abatacept has a different mode of action in addition to its blocking effect on T cell costimulation in humans.

The observation that abatacept is capable of decreasing disease activity in the absence of CD4^+^ T cells does not contradict the dogma that the mode of action of abatacept is mediated through blockade of costimulation and thereby activation of (naive) CD4^+^ T cells. However, our results do indicate that abatacept has another mode of action in addition to its effects on costimulation. A limitation of our study is that we did not elucidate the mechanism responsible for the inhibitory effect of abatacept on arthritis. In this respect, abatacept is no different from other DMARDs, such as methotrexate, for which the exact mode of action also has not been elucidated. Nonetheless, it would be interesting to delineate these additional modes of action [17], as it could allow for a more refined targeted therapy and additional insights into the aetiological pathways of disease.

Recently, Rozanski et al*.* described that CD28 serves as a survival factor for long-lived plasma cells. Loss of CD28 or B7.1 (CD80) and B7.2 (CD86) caused significant loss of long-lived plasma cells, resulting in decreased antibody titres [18]. As abatacept prevents the binding of CD28 to CD80 and/or CD86, this survival signal could be abrogated and lead to loss of plasma cells and consequently a decrease in antibody titres. Indeed, this would be in line with our observation of decreased antibody levels after abatacept treatment and reduction of clinical scores, as well as our observation of decreased antibody production by cultured spleen and bone marrow cells from CD4^+^ T cell–depleted mice treated with abatacept. Likewise, in the BXD2 mouse model of autoimmune disease, it has been reported that elevated expression of activation-induced cytidine deaminase (AID) in recirculating follicular CD86^+^ B cells and increased germinal centre activity are associated with the production of autoantibodies [19]. Treatment with CTLA-4-Ig resulted in normalisation of AID expression in the B cells and suppression of IgG autoantibodies, which could explain the decrease in IgG titres we observed after abatacept treatment in the absence of CD4^+^ T cells.

The CD4^+^ T cell–independent effect of abatacept could also be explained by the induction of nitric oxide synthase or indoleamine 2,3-dioxygenase (IDO) by APCs [20–22]. IDO is an enzyme that degrades the essential amino acid tryptophan, resulting in local depletion of tryptophan [23], which leads to cell cycle arrest [24, 25] and thereby to inhibition of T cell proliferation and expansion of the immune response [26–28]. IDO has been implicated in disease aetiology, as, for example, it has been reported that CTLA-4 on regulatory T cells can induce IDO in APCs [29] but regulatory T cells from RA patients failed to induce such expression owing to low CTLA-4 expression [30]. In addition to the suppressive effect of IDO on proliferating effector T cells, IDO-expressing dendritic cells are able to promote the activation of regulatory T cells [31] and the differentiation of naive T cells to regulatory T cells [23, 32], which could explain the inflammation-suppressing effects of abatacept. However, Davis et al. [33] reported inhibition of naive and memory T cell proliferation and effector function in the absence of IDO induction, indicating that abatacept could also have other mechanisms of action.

Abatacept could also have an effect on osteoclast precursors, explaining the anti-erosive effects of abatacept [34, 35], or on monocytes by modulating their migratory capacity [36]. Direct effects on macrophages have also been described resulting in decreased cytokine production and reduction of the inflammatory reaction [37–40], which could account for the beneficial effects in the treatment of RA.

## Conclusions

Abatacept is thought to block costimulation of T cells by blocking the interaction between CD28 and B7, resulting in inhibition of T cell activation. However, abatacept reduced disease progression and activity in the absence of CD4^+^ T cells in the CIA mouse model, indicating that abatacept can exert its action in established arthritis independently of its effects on CD4^+^ T cell activation. Because a head-to-head comparison of anti-TNF and abatacept treatment resulted in comparable efficacy with a similar time course, it is tempting to speculate that abatacept could have an effect on other cell types in addition to T cells in human RA as well.

## Abbreviations

AID: activation-induced cytidine deaminase
APC: antigen-presenting cell
AU: arbitrary units
CFA: complete Freund’s adjuvant
CIA: collagen-induced arthritis
CII: collagen type II
CTLA-4: cytotoxic T-lymphocyte–associated protein 4
DMARD: disease-modifying anti-rheumatic drug
ELISA: enzyme-linked immunosorbent assay
HRP: horseradish peroxidase
IDO: indoleamine 2,3-dioxygenase
IFA: incomplete Freund’s adjuvant
IgG: immunoglobulin G
IL: interleukin
LN: lymph node
mAb: monoclonal antibody
ns: not significant
NSAID: non-steroidal anti-inflammatory drug
PBS: phosphate-buffered saline
RA: rheumatoid arthritis
TNF: tumour necrosis factor
